# ‘She was referred from one hospital to another’: evidence on emergency obstetric care in Karnataka, India

**DOI:** 10.1186/1753-6561-6-S5-P15

**Published:** 2012-09-28

**Authors:** HY Vijayashree, Maya Annie Elias, Mamata R Patil, MH Anil, MR Raveesha, Narayanan Devadasan, Arima Mishra, Patrick Van Dessel

**Affiliations:** 1 Institute of Public Health, Bangalore, India; 2 Institute of Tropical Medicine, Antwerp, Belgium

## Introduction

Launching of the the National Rural Health Mission (2005) has brought a sharp policy attention to maternal health with its several initiatives such as (a) cash and material benefits to pregnant women; (b) strengthening health institutions especially of First Referral Units (FRUs); and (c) conducting regular maternal death audits to ensure quality Emergence Obstetric Care (EmOC). In spite of these efforts, the decline in maternal mortality ratio is rather slow and not achieved as per the MDG 5 goal. This study was undertaken to understand why despite infusion of such vast resources into the health system, timely and quality EmOC services have not been implemented.

## Methods

We conducted in-depth interviews with users of EmOC services, semi-structured interviews with key actors of both private and public health system and focus group discussions among field health workers. Data were collected from July to December 2011 in two districts of Karnataka each in north and south Karnataka, as part of a larger multi-country research project ‘Health System Stewardship and Regulations in Vietnam, India and China (HESVIC)’.

Users were selected (n = 10) through purposive sampling using outcomes criteria such as bad outcomes (n = 6) [maternal deaths (4), still births (2)] and good outcomes (n = 4) to understand their experiences of EmOC provision. Ethical clearance was obtained and written consent was sought from all respondents. Data was collected according to the saturation principle. Interviews were audio recorded and transcribed verbatim. Thematic analysis was done and at certain stages critical discourse analysis was employed to examine connections across several texts (interviews, grey material etc.) and contexts.

## Results

All interviewed users were living below the poverty line belonging to scheduled caste or tribe and had completed primary education, except one user who had a good outcome.

### Unpredictable EmOC services

While there is a rise in institutional deliveries, upgraded FRUs have not been able to ensure 24×7 services. Though all interviewed users were registered with auxiliary nurse-midwives (ANM) and were given advice about nutrition and immunisation, they opined that government hospitals can be relied for antenatal visits and for normal deliveries, but they are unpredictable when it comes to provide EmOC services. Users were not sure where and when EmOC cases are managed successfully and when they would be referred out. Below is an excerpt depicting the uncertainty of EmOC provision at government hospitals: *“We hesitate to go to Government hospital because of their usual advice to go and get the delivery done elsewhere”.*

### Multiple referrals for averting risk

All bad outcome cases (n = 6) had experienced multiple referrals both in public and private health facilities. There was a time lapse of minimum 10 hours to maximum 32 hours to receive required EmOC services. They were referred to four different hospitals in this time span. The highest health facility most commonly visited was the district hospital. Maternal deaths had taken place in the process of being hopped from one hospital to another even though they had made serious efforts to access EmOC services both in private and public health facilities. Ensuring the availability of blood was seen as the responsibility of the patient’s relatives, without which specialists would refer them out.

While lack of resources definitely has been a major reason for referral, a tendency to avoid high risk cases mainly for the fear of facing the maternal death audits and blame that has led to a number of unnecessary referrals. There is tendency of transferring of such blame along the hierarchy. For instance, health workers often claim that no deaths happen in the ‘field’, these happen in hospitals, hospital officials would like to say ‘deaths often happen in transit not in hospitals’. A specialist explains, *“better to refer the patient*, *than get caught up in unnecessary problems of audit and the humiliation that we experience”.*

### Provision of EmOC service by ‘chance’ and ‘luck’

Good outcomes (n = 4) were due to *‘good luck’* or *‘chance’* where all the necessary resources were made available at that particular time due to the interventions of caste-based organisations, of rickshaw-driver unions, or of a local practitioner. This uncertainty is because many FRUs do not function or partially function because of lack of availability of specialists. Either sanctioned posts are not filled up or when they are filled, specialists are either on long leave or running private practice. In some instances, specialists are available but their services are mis-utilised for general out-patient department and administrative work, which makes specialist services unavailable round the clock at FRUs. NRHM initiatives like training of MBBS doctors in anaesthesia and caesarean section to redress the specialist shortage has had a limited success due to the perceived lack of confidence and team support.

Other factors such as individual motivations of providers, personal equation with private doctors, ability to negotiate with providers and access to local political support also determined the access to EmOC services.

## Discussion

While physical resources like buildings and financial resources have improved under NRHM, human resources particularly of specialists remain a critical bottleneck and prove an obstacle to ensure EmOC care when needed. Training of medical doctors in providing EmOC with more emphasis on practical training may restore their confidence in providing EmOC services, thus address the issue of shortage of specialists at FRUs. Maternal death audits should be conducted in a conducive environment with an aim to strengthen the health system to prevent maternal deaths rather than a faultfinding exercise. The practice of unnecessary referrals calls for a stricter accountability mechanisms not only in public sector but also in private sector. Further, we recommend a robust referral mechanism with stricter adherence to prescribed protocol such as monitoring of partogram. Government should focus on making blood storage units functional. Otherwise women will continue to face limited access to EmOC services.

## Competing interests

None declared.

## Funding statement

This study was funded by the European Commission funded HESVIC project (2009-2012).

